# Deciphering the Oncogenic Role of VPS28 Modulated by miR-491-5p in Breast Cancer Cells Using In Silico and Functional Analysis 

**DOI:** 10.3389/fmolb.2021.634183

**Published:** 2021-07-30

**Authors:** Wenjie Shi, Daojun Hu, Yu Xing, Rui Zhuo, Qiufeng Lao, Hui Liu, Weiyi Pang

**Affiliations:** ^1^ School of Public Health, Guilin Medical University, Guilin, China; ^2^ Department of Clinical Laboratory, Xinhua Hospital Chongming Branch, Shanghai, China; ^3^ Department of Breast Surgery, Guilin TCM Hospital of China, Affiliated to Guang Xi University of Chinese Medicine Guilin, Guilin, China

**Keywords:** miR-491-5p, breast cancer, proliferation, invasion, migration, VPS28

## Abstract

Vacuolar protein sorting–associated protein 28 (VPS28), one of the four cytosolic proteins comprising the endosomal sorting complex required for the transport I (ESCRT-I) component, has been reported to be linked to various cancers. However, less evidence is available regarding the involvement of VPS28 in breast cancer. To this end, this study focused on exploring the function of VPS28 in breast cancer cells using the *in silico* analysis. VPS28 expression pattern data in breast cancer tissues were collected using the Cancer Genome Atlas (TCGA) and Clinical Proteomic Tumor Analysis Consortium (CPTAC) databases and analyzed to assess the association of VPS28 with breast cancer prognosis. The elevated VPS28 expression was found in breast cancer tissues and was associated with a poor prognosis (*p* < 0.001). A higher VPS28 expression indicated a short survival duration (HR = 2.43; 95% CI: 1.44–4.1; *p* < 0.001). The CCLE database showed that VPS28 was expressed in breast cancer cell lines. The upstream targets of VPS28 were identified using the mirDIP, starBase, and TargetScan online tools. The correlation and binding relationship between miR-491-5p and VPS28 was analyzed. *VPS28* or miR-491-5p gain and loss of function experiments were performed to verify their potential effect on the biological functions of breast cancer cells. Knockdown of VPS28 was shown to suppress the biological functions and enhance the apoptosis of breast cancer cell lines. Micro RNA-491-5p, identified as a posttranscriptional regulator of VPS28, was downregulated in breast cancer tissues. In contrast to the miR-491-5p inhibitor, the miR-491-5p mimic could suppress the migration, wound healing ability, and proliferation, while accelerating apoptosis. However, co-transfection of VPS28 and miR-491-5p counteracted the effect of the miR-491-5p mimic on breast cancer cell functions. Thus, our *in silico* analysis demonstrates that miR-491-5p can suppress breast cancer progression by attenuating the expression of VPS28.

## Introduction

Breast cancer is the most common form of cancer affecting women and is characterized by a high relapse rate and a poor prognosis (Anastasiadi et al., 2017; Kolak et al., 2017). Although great advances have been made in the screening and diagnosis of breast cancer, younger women (under 40 years) are increasingly being diagnosed with the disease (Rossi et al., 2019). This indicates that sex, estrogen, family history, gene mutations, and unhealthy lifestyle are important risk factors for breast cancer development, in addition to aging (Sun et al., 2017a). The prognosis for breast cancer is greatly determined by the time of initial diagnosis, and those diagnosed at an early stage can often be successfully cured (Wörmann, 2017). Nevertheless, *de novo* tumor cells and resistance to anticancer agents pose great challenges for the treatment of breast cancer (Yu et al., 2015). Progress in identifying therapeutic strategies has been hampered by the insufficient understanding of the complex tumor microenvironment. Therefore, a better understanding of the mechanisms implicated in tumor progression is urgently needed.

Mounting evidence supports the role of certain proteins and micro-RNAs (miRNAs) in breast cancer progression (Li et al., 2017; Rahman et al., 2019). Vacuolar protein sorting–associated protein 28 (VPS28), an endosomal sorting complex required for transport I (ESCRT-I) component, is required for cell survival. VPS28 was shown to regulate the mitotic spindle organization by interacting with the Gβγ, EG5, and TPX2 proteins (Dionisio-Vicuña et al., 2018). Nevertheless, whether VPS28 regulates plasma membranes and cell apoptosis in the context of cancer remains unclear. VPS28 has previously been reported to improve the uptake of anti–miR-21 in parental SKHEP1 cells (Wagenaar et al., 2015). However, little information exists regarding the possible interaction between VPS28 and miRNAs. Micro-RNAs are a class of noncoding RNAs capable of regulating the expression of protein-coding genes (Correia De Sousa et al., 2019). The role of miR-491-5p as a tumor suppressor has been documented in many types of tumor (Chen et al., 2018; Lu et al., 2019). In gastric cancer, Foxi1/miR-491-5p/Wnt3a/β-catenin signaling has been proposed to be a potential therapeutic avenue for cancer treatment (Sun et al., 2017b). In ovarian cancer, miR-491-5p has been shown to induce apoptosis by overriding the antiapoptotic activities of BCL-X (L) and MCL1 (Denoyelle et al., 2014). Data from previous studies showed that miR-491-5p was associated with breast cancer progression (Tan et al., 2019; Guo et al., 2021). However, more evidence is required to gain a comprehensive understanding of the role of miR-491-5p in breast cancer.

In this study, public databases (including TCGA, CPTAC, and mirDIP) were searched to elucidate the role of VPS28 in breast cancer tissues and cells. By further tracking its upstream target, we identified miR-491-5p as a posttranscriptional regulator of VPS28 in breast cancer cell lines. By monitoring the proliferation, migration, and apoptosis of transfected cells, we found that VPS28 upregulation was involved in tumor progression by downregulating the miR-491-5p expression.

## Materials and Methods

### Bioinformatic Analysis

We used R software to obtain breast cancer count data from the TCGA databases (https://genome-cancer.ucsc.edu/). After standardization, these raw data were converted into transcripts per million (TPM) data for subsequent analysis. In parallel, the expression of VPS28 in different cancer cells, including breast cancer cell lines, was verified using the CCLE database (https://portals.broadinstitute.org/ccle), which stores genomics data of more than 1,000 cancer cell lines. The mRNA expression pattern of VPS28 in pan-cancers, as well as in cell lines, was identified using the TCGA database. We also used the TCGA database to further verify the expression of VPS28 in different breast cancer subtypes. Meanwhile, single- and multi-batch Cox regression analyses were used to determine the independent risk of various mRNAs in breast cancer prognosis. Next, a survival curve for patients with different VPS28 expression levels was calculated using the survival package. The CPTAC database was used to verify VPS28 protein expression levels. The mirDIP (http://ophid.utoronto.ca/mirDIP/index.jsp#r), starBase (http://starbase.sysu.edu.cn/), and TargetScan (http://www.targetscan.org/vert_72/) tools were then used to predict the upstream targets of VPS28. The predicted miRNAs were verified using the TCGA database, and the associated survival curve was generated using the survival package.

### Cell Culture

The mammary epithelial cell line (MCF10A) and breast cancer cell lines (MDAMB231, BT474, MCF7, T47D, and MDAMB436) were purchased from American Type Culture Collection (ATCC, United States). RPMI 1640 culture medium containing 10% fetal bovine serum, 1% glutamine, and 1% antibiotic/antifungal solution was used to culture cells at 37°C, 5% CO_2_.

### Cell Transfection

The VPS28 short hairpin oligonucleotide (shRNA, 1 μg) and its negative control (sh-NC, 1 μg) were designed and synthesized by Gene Seed Biotechnology Co., Ltd. (Guangzhou, China). The miR-491-5p mimic and inhibitor (100 nM) and the corresponding negative control (mimic-NC and inhibitor-NC, 100 nM) were obtained from RiboBio Co., Ltd. (Guangzhou, China). The pcDNA3.1 plasmid (2 μg), used to generate the VPS28 construct, was provided by Thermo Fisher Scientific (Waltham, MA, United States). Transfection of the T47D and MCF7 cell lines was performed using Lipofectamine 3,000 (Invitrogen) in accordance with the manufacturer’s instructions. All transfected cells were cultured for 48 h prior to use in experiments.

### Quantitative Reverse Transcription–Polymerase Chain Reaction

Forty-eight hours following transfection, the T47D and MCF7 cells were subjected to total RNA extraction using the Trizol reagent (Invitrogen) extraction kit. The concentration and purity of extracted RNA was determined by a NanoDrop 2000 spectrophotometer (Thermo Scientific, United States). RNAs with OD260/OD280 values in the 1.7–2.1 range were qualified for cDNA transcription using a reverse transcription kit (Bioer Technology Co., Ltd., Hangzhou, China). Quantitative RT-PCR was performed using the qPCR LightCycler® 480 system (Roche Diagnostics), with SYBR® Premix Ex Taq™ II (Takara, Dalian, China) and TaqMan universal Master Mix II (Life Technologies Corporation, Carlsbad, CA, United States), according to the manufacturer’s instructions. The following PCR reaction conditions were used: 95°C for 30 s, 95°C for 10 s, 60°C for 30 s, and 70°C for 34 s for a total of 40 cycles. The expression of VPS28 and miR-491-5p was determined using GAPDH and U6 as reference, respectively. Relative expression was calculated using the 2^−ΔΔCt^ method. Experiments were performed in triplicate. Primer sequences are listed in Table 1.

**TABLE 1:** Primer sequences for qRT-PCR.

Name of primer	Sequences
*miR-491-5p*-F	CCG​GAA​TTC​TTT​CTG​GGT​AGC​CTT​TAG​C
*miR-491-5p*-R	CGC​GGA​TCC​TCA​AAT​AGC​CAT​CCT​AGA​CT
U6-F	CTC​GCT​TCG​GCA​GCA​CAT​ATA​CT
U6-R	ACG​CTT​CAC​GAA​TTT​GCG​TGT​C
*VPS28*-F	TCA​TAC​AGC​TCC​GGC​TTG​TT
*VPS28*-R	GGT​TGG​AAC​CTC​TGT​GCT​CC
GAPDH-F	GTC​GAT​GGC​TAG​TCG​TAG​CAT​CGA​T
GAPDH-R	TGC​TAG​CTG​GCA​TGC​CCG​ATC​GAT​C

### Western Blotting

RIPA lysis buffer (Beyotime, Shanghai, China) was utilized to extract total protein from T47D and MCF7 cells. The extracted protein concentration was determined using the BCA protein assay kit II (BIO-RAD, Hercules, CA, United States). A BCA standard curve was constructed using the following standard sample dilutions: 20, 15, 10, 5, 2.5, and 0 μg/μL. The total volume was calculated accordingly. After that, the protein (25 μL) and standard (25 μL) samples were added to wells of a 96-well plate, in duplicate. Next, 200 μL of solution was added to each well, followed by gentle shaking and incubation at 37°C for 30 min. After cooling to room temperature, the samples were subjected to absorbance measurement at 495 nm to determine the protein concentration. The proteins were separated by 10% SDS-PAGE gel and transferred onto a 0.22-μm PVDF membrane, before blocking with skim milk. Primary antibodies against VPS28 (Abcam, ab154793, 1/1,000) or *β*-actin (Abcam, ab115777, 1/200) were then added, prior to incubation at 4°C overnight. The membranes were then washed with TBST thrice for 10 min and incubated with HRP-labeled goat anti-rabbit IgG (Abcam, ab205718, 1/2000) for 1 h. Membranes were developed using the ECL reagent (Millipore, Plano, TX, United States) and analyzed using ImageJ software. The relative expression level of target protein = grey value of target protein/grey value of *β*-actin.

### 3-(4,5-dimethylthiazol-2-yl)-2,5-Diphenyl Tetrazolium Bromide Assay

Cells in the logarithmic phase were inoculated into a 96-well plate at a density of 1 × 10^4^ cells/well and cultured for 0, 12, 24, 48, or 72 h at 37°C with 5% CO_2_. Next, 20 μL of MTT was added to the culture medium, and the samples were incubated for 4 h at 37°C, before adding 150 μL DMSO and incubating for a further 10 min. Absorbance at 450 nm was set to ordinate, while time was set to abscissa, for the purpose of MTT curve construction. The results were averaged over three independent experiments.

### Clone Formation Assay

T47D and MCF7 cells in the logarithmic phase were seeded into a six-well plate at 1 × 10^3^ cells/well and cultured in an incomplete culture medium for 2–3 weeks. The cells were then fixed with 4% paraformaldehyde (PFA) for 30 min before staining with 0.5% crystal violet staining solution at room temperature for 30 min. Cell clones were quantified under the microscope with >50 cells representing one clone. The results were averaged over three independent experiments.

### Cell Scratch Assay

1 × 10^5^ cells/well resuspended in 100 µL media were plated out in 6-well plates. Once cells reached 95% confluency, the tip of a 10-µL pipette was used to make a scratch in the cell layer, making sure that each scratch had the same width. The plates were then washed with PBS and cultured in a complete culture medium containing 1% FBS at 37°C with 5% CO_2_. The cell scratch width was observed under an inverted microscope 0 and 24 h after the cell scratch was made.

### Transwell Assay

T47D and MCF7 cells were resuspended in a serum-free culture medium at 2 × 10^4^ cells/mL. The cell suspension (200 µL) was added to the serum-free transwell upper chamber coated with Matrigel. The lower chamber was filled with the 500 µL complete culture medium containing 10% FBS. The transwell system was incubated at 37°C for 24 h with5% CO_2_. After that, the cells in the lower chamber were stained with 0.1% crystal violet solution for 15 min and counted under a light microscope.

### Annexin-V/PI Staining for Apoptosis

Cells in each group were treated with 0.25% trypsin and pelleted by centrifugation. After three washes with PBS, the cell pellets were stained using the Annexin V/PI staining kit (BD Biosciences, MA, United States), according to the manufacturer’s instructions. Cell apoptosis was assessed using the cytoFLEX LX flow cytometer (Beckman Coulter Electronics, Jiangsu, China) and CytExpert software.

### Dual Luciferase Reporter Gene Assay

The TargetScan and starBase online prediction tools were applied to predict the binding sites of VPS28 and miR-491-5p, respectively. The wild-type and mutant sequences of the binding sites were designed. The 3′UTR region of VPS28, containing the targeting sites for miR-491-5p, was then amplified by PCR and cloned into the pGL3 luciferase vector (Promega, Madison, WI, United States). The QuikChange II Site-Directed Mutagenesis Kit (Agilent Technologies, Santa Clara, CA, United States) was used to generate the mutant VPS28 3′UTR. The wild-type and mutant VPS28 3′UTRs were co-transfected with either the miR-491-5p mimic or the NC mimic into HEK293T cells. Firefly and Renilla luciferase activities were determined approximately 48 h after transfection.

### Statistical Analysis

Data analysis was performed using GraphPad Prism 6.0 (GraphPad Inc., San Diego, CA, United States) and R (version 3.6.1) software. The parameters related to disease prognosis were determined using the Cox regression analysis, and the survival differences among groups were analyzed using the log-rank test. Data not complying with a normal distribution were analyzed using the Wilcoxon signed rank test, while analyses of data complying with a normal distribution employed the Mann–Whitney *U* test. A comparison between two groups was performed using the Student’s *t*-test, while multiple group comparisons were analyzed using the one-way ANOVA with Tukey’s multiple comparison test for the *post hoc* analysis. Three duplicates were set up for each group, with *p* values less than 0.05 indicating a significant difference.

## Results

### Vacuolar Protein Sorting–Associated Protein 28 Upregulation in Breast Cancer Tissues Is Associated With a Poor Prognosis

**FIGURE 1:**
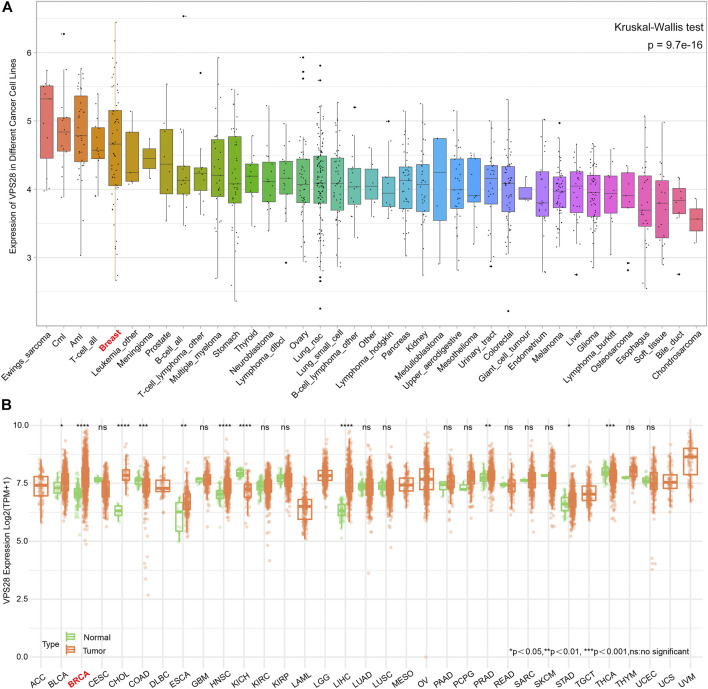
The mRNA expression of VPS28 in pan-cancer cell lines and tissues. The mRNA expression pattern of VPS28 in pan-cancer cell lines according to data collected from the CCLE database **(A)** and in pan-cancer tissues from the TCGA database **(B)**.

**FIGURE 2:**
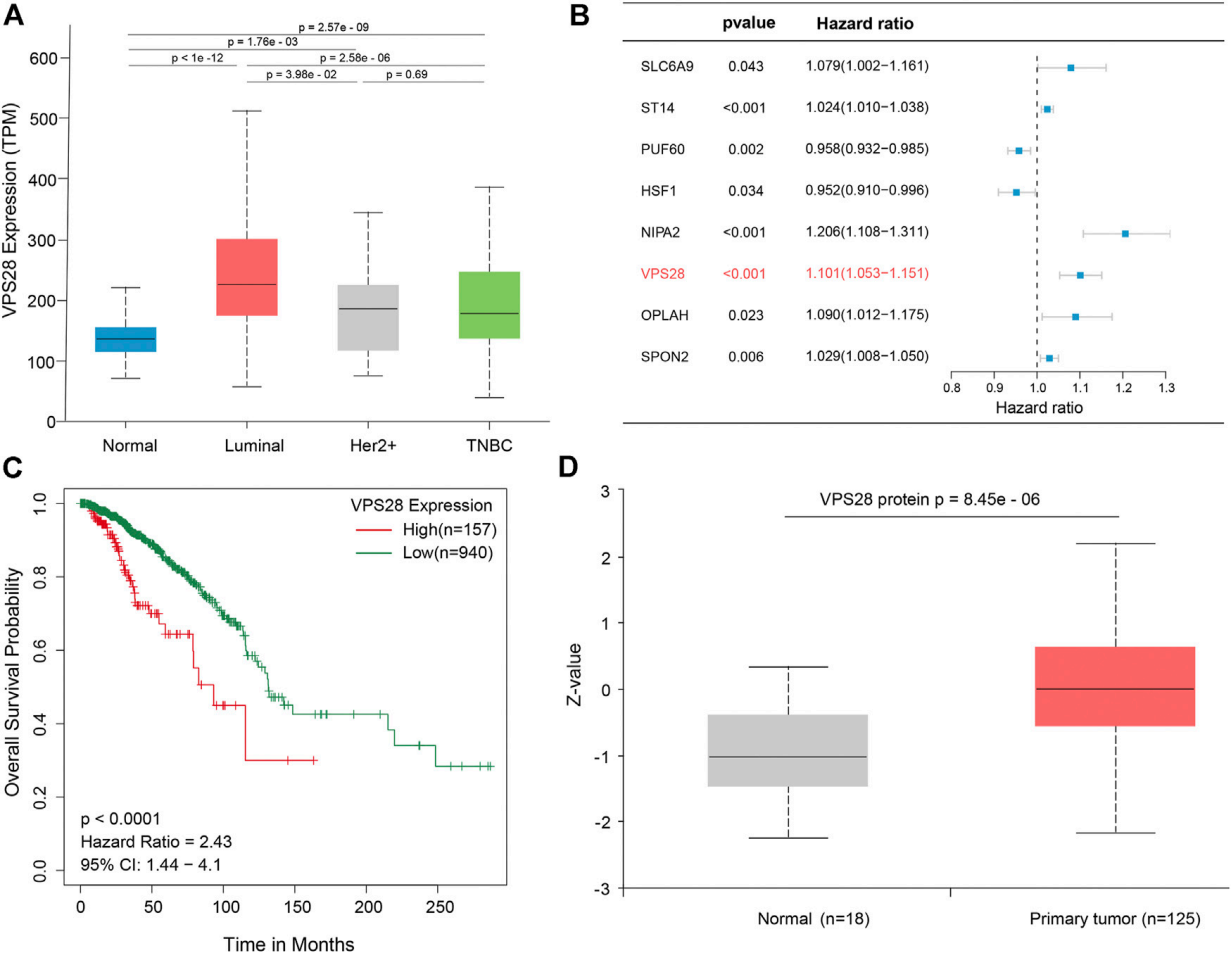
VPS28 expression in different subtypes of breast cancer, according to the TCGA database **(A)**. Batch multi-Cox regression analysis for OS showed that eight genes represented independent risk factors for breast cancer, including *SLC6A9*, *ST14*, *PUF60*, *HSF1*, *NIPA2*, *VPS28*, *OPLAH*, and *SPON2* **(B)**. K-M analysis shows that high expression VPS28 was associated with a poor prognosis of breast cancer patients **(C)**. VPS28 protein expression levels in breast cancer tissues and normal mammary tissues according to the CPTAC database **(D)**.

**FIGURE 3:**
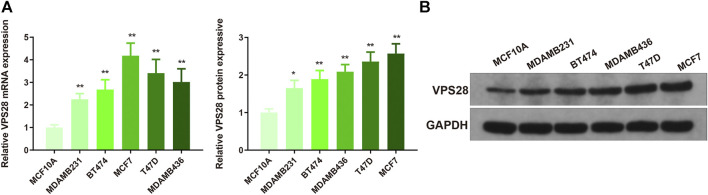
*VPS28* mRNA and protein expression levels in normal breast cell line and breast cancer cell lines. *VPS28* mRNA and protein expression levels in MCF-10A and breast cancer cells (MDAMB231, BT474, MCF7, T47D, and MDAMB436) determined by qRT-PCR and Western blotting **(A**,**B)**; * *p* < 0.05, **p < 0.01, *n* = 3.

The mRNA expression of VPS28 in breast cancer cell lines was tightly low than four types in total 39 cell lines (Figure 1A, *p* = 9.7e-16). In addition, the VPS28 mRNA expression in breast cancer tissue samples (*n* = 1,099) was higher than that in normal mammary tissue samples (*n* = 113; Figure 1B, *p* < 0.001). Moreover, the expression of VPS28 varied among cancer subtypes, of which the Luminal subtype displayed the highest level of the VPS28 expression (Figure 2A). The Cox regression analysis of overall survival (OS) for 1,097 patients, with complete 25-year follow-up information, showed a certain relationship between the VPS28 expression level and the poor prognosis of breast cancer patients (HR = 1.101; 95% CI: 1.053–1.151; Figure 2B, *p* < 0.001). The K–M analysis showed that tumors over-expressing VPS28 (over the third quantile) present a shorter OS (HR = 2.43; 95% CI: 1.44–4.1; Figure 2C, *p* < 0.001). Upregulated VPS28 protein expression was also verified using the CPTAC database (Figure 2D, *p* < 0.001). Analysis of MCF10A and human breast cancer cells (MDAMB231, BT474, MCF7, T47D, and MDAMB436) by qRT-PCR and Western blotting showed that T47D and MCF7 cells had the highest VPS28 expression levels compared with the other cell lines tested (Figures 3A,B, * *p* < 0.05 and **p < 0.01). Therefore, T47D and MCF7 cell lines were selected for further experiments.

### Suppression of Vacuolar Protein Sorting–Associated Protein 28 Expression Attenuates Breast Cancer Progression

**FIGURE 4:**
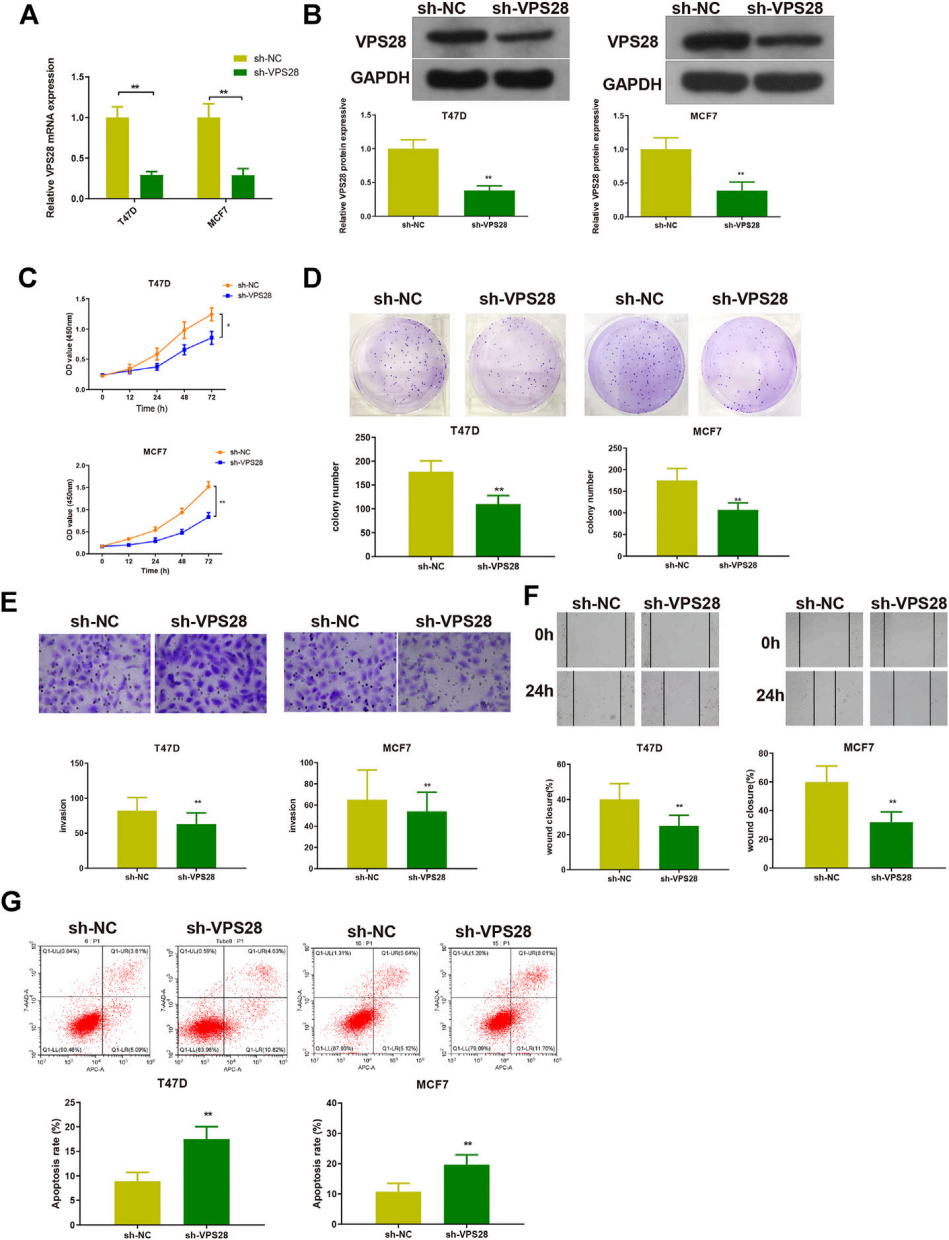
The effect of VPS28 on the proliferation, migration, invasion ability, and apoptosis of breast cancer cells was determined after cells were transfected with sh-NC or sh-VPS28. The transfection efficiency was verified by qRT-PCR **(A)** and Western blotting **(B)**. After cell transfection, the cell viability **(C)**, and clone formation **(D)**, migration **(E)**, and invasion **(F)** abilities were measured. Cell apoptosis was determined by flow cytometry **(G)**. *p < 0.05, **p < 0.01, *n* = 3.

The T47D and MCF7 cell lines were transfected with sh-VPS28. The transfection efficiency, measured by qRT-PCR and Western blotting, was satisfactory (Figures 4A,B, **p < 0.01). Measurement of cell viability, migration, and invasion showed that VPS28 knockdown could reduce the viability and proliferation (Figures 4C,D, * *p* < 0.05, **p < 0.01), as well as the migration and invasion capacities of both T47D and MCF7 cell lines (Figures 4E,F, * *p* < 0.01). Flow cytometry showed that the extent of apoptosis was higher in the sh-VPS28 group than that in the sh-NC group (Figure 4G, *p* < 0.05).

### Micro Ribo Nucleic Acid-491-5p Negatively Regulates Vacuolar Protein Sorting–Associated Protein 28 in Breast Cancer Cells

**FIGURE 5:**
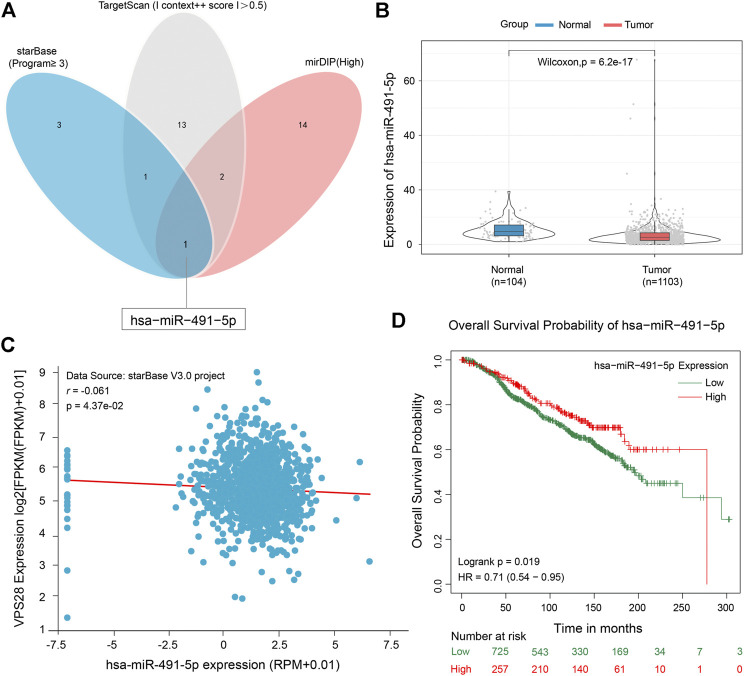
Micro RNA-491-5p can negatively target VPS28. A Venn diagram of the upstream targets of VPS28 as assessed by starBase, TargetScan, and mirDIP **(A)**. The expression pattern of miR-491-5p in breast cancer using TCGA data **(B)**. The correlation between miR-491-5p and VPS28 levels was analyzed in breast cancer tissues **(C)**. The effect of miR-491-5p on the overall breast cancer patient survival rate according to the TCGA database **(D)**.

To clarify the working mechanism of VPS28 in breast cancer cells, we used the mirDIP, starBase, and TargetScan tools to search for its upstream targets. We identified miR-491-5p as a binding partner of VPS28 (Figure 5A). The TCGA database showed that miR-491-5p was downregulated in breast cancer tissues (*n* = 1,103), compared to normal mammary tissues (*n* = 104) (Figure 5B, *p* < 0.001). Analysis of starBase data revealed that miR-491-5p levels expressed an inversely correlated trend with VPS28 levels in breast cancer cells (Figure 5C). Additionally, we also found that breast cancer patients with lower miR-491-5p expression levels had shorter overall survival periods (HR = 0.71; 90% CI: 0.54–0.95; *p* = 0.019, Figure 5D).

**FIGURE 6:**
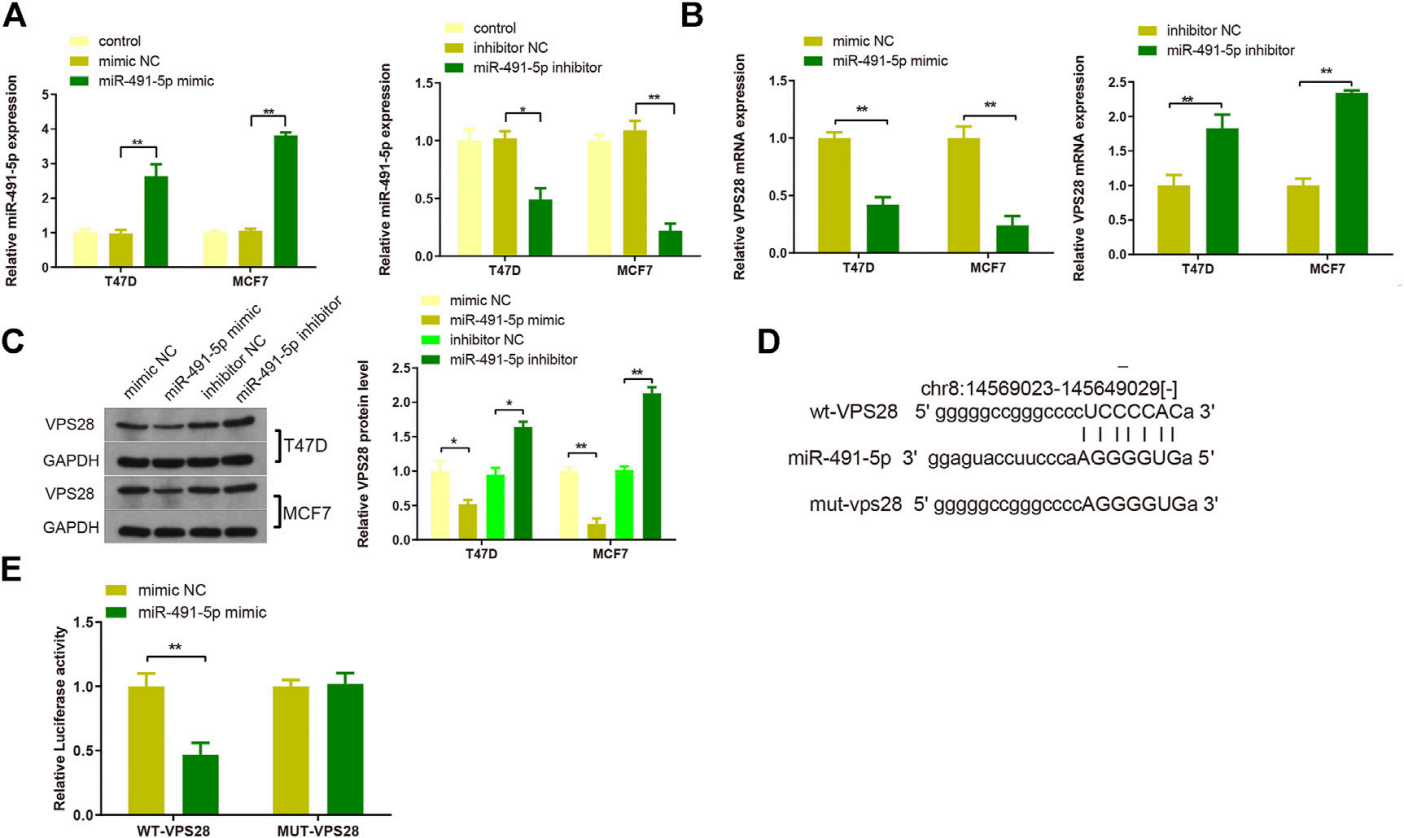
The transfection efficiency of T47D and MCF7 cells with miR-491-5p was determined by qRT-PCR **(A)**. mRNA and protein expressions of VPS28 were detected by qRT-PCR **(B)** and Western blot **(C)**. The predicted binding site of miR-491-5p and *VPS28* 3′UTR as determined by TargetScan **(D)**. The binding of miR-491-5p to *VPS28* was determined using the dual luciferase reporter assay **(E)**. **p < 0.01, ***p < 0.01, *n* = 3.

T47D and MCF7 cell lines were then transfected with either a miR-491-5p mimic or a miR-491-5p inhibitor, and the transfection efficiency was verified by qRT-PCR (Figure 6A, **p < 0.01). Western blotting and qRT-PCR analyses of cells transfected with the miR-491-5p mimic or the miR-491-5p inhibitor showed that VPS28 mRNA and protein expression levels were suppressed in the miR-491-5p mimic group but increased in the miR-491-5p inhibitor group (Figures 6B,C, **p < 0.01). The TargetScan web server predicted the binding sequences of VPS28 and miR-491-5p, based on which we constructed wild-type and mutant VPS28 3′UTRs (Figure 6D). The dual luciferase reporter gene assay showed that the miR-491-5p mimic significantly suppressed the luciferase intensity of wild-type VPS28 (Figure 6E, **p < 0.01).

### Micro Ribo Nucleic Acid-491-5p Suppresses Breast Cancer Progression by Down-regulating Vacuolar Protein Sorting–Associated Protein 28

**FIGURE 7:**
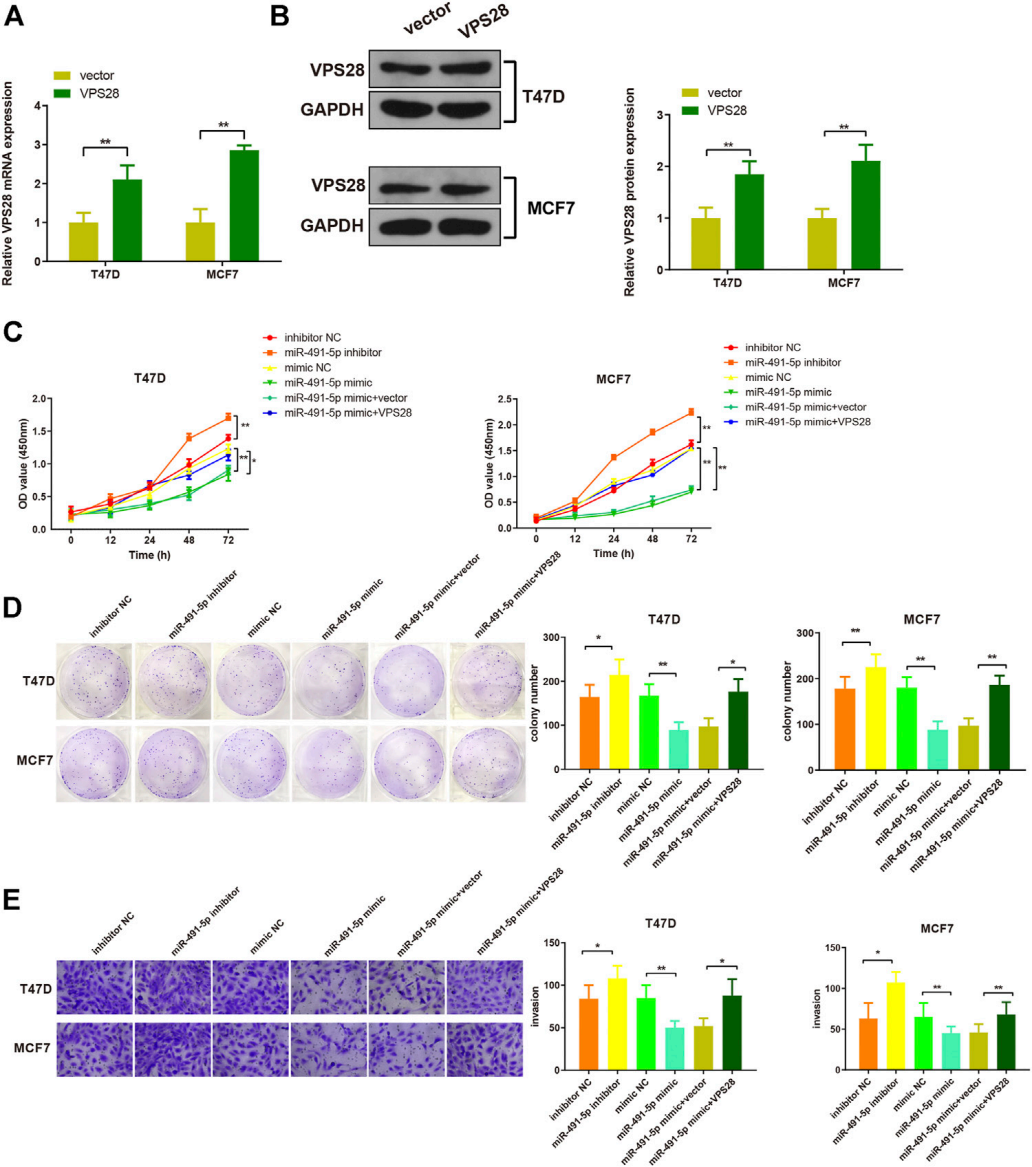
Micro RNA-491-5p suppresses the proliferation and invasion of breast cancer cells through the regulation of *VPS28*. VPS28 overexpression was achieved in breast cancer cells whose transfection efficiency was determined by qRT-PCR **(A)** and Western blotting **(B)**. Cell viability, proliferation, and migration ability were detected by MTT **(C)**, clone formation assay **(D)**, and scratch assay, respectively **(E)**. *p < 0.05, **p < 0.01, *n* = 3.

**FIGURE 8:**
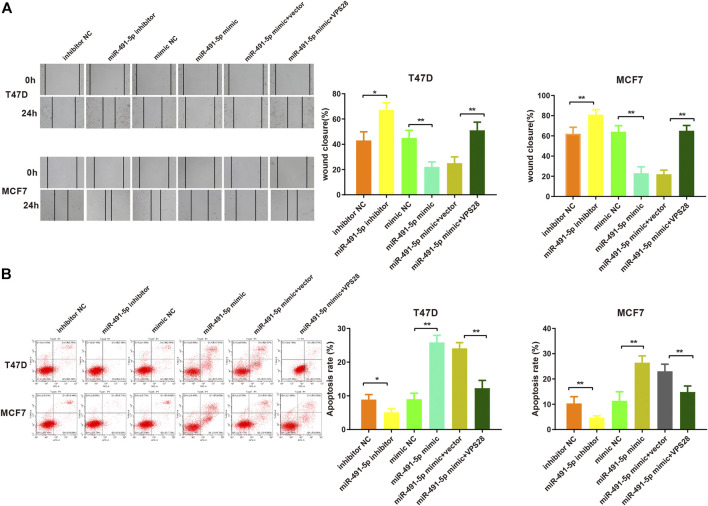
Micro RNA-491-5p inhibits the invasiveness and apoptosis of breast cancer cells by regulating VPS28. Cell invasiveness was measured using the transwell assay **(A)**, while cell apoptosis was determined by flow cytometry **(B)**. **p < 0.01, ***p < 0.001, *n* = 3.

Considering the regulatory effect of miR-491-5p on VPS28, we first transfected a VPS28-overexpressing plasmid into breast cancer cells. Western blotting and qRT-PCR assays showed that the transfection efficiency was satisfactory (Figures 7A,B, *p < 0.05, **p < 0.01). The MTT, clone formation, cell scratch, and transwell assays showed that the miR-491-5p inhibitor could increase the proliferation, migration, and invasion ability of breast cancer cells, while the reverse pattern was found in the miR-491-5p mimic group (Figures 7C–E). In addition to that, the suppressive effect of the miR-491-5p mimic on cell viability and migration could be abolished by VPS28 overexpression (Figure 8A, *p < 0.05, **p < 0.01). Inhibition of miR-491-5p was also shown to suppress cell apoptosis, while miR-491-5p overexpression had the opposite effect. Furthermore, the overexpression of VPS28 was able to suppress the effect of miR-491-5p overexpression on cell apoptosis (Figure 8B).

## Discussion

Bioinformatic analysis of databases such as TCGA, CPTAC, and CCLE highlighted the increased expression of VPS28 in breast cancer tissues and cell lines. In this study, we aimed to examine the role of VPS28 in breast cancer progression by measuring its effect on the proliferation, migration, invasion, and apoptosis of two breast cancer cell lines.

VPS28 belongs to the ESCRT family of proteins, which is largely responsible for membrane scission or sealing by cooperation with the ATPase VPS4 (Vietri et al., 2020). Cellular membranes are of critical importance in many cellular processes, supporting cytokinesis, autophagy, and membrane repair (Henne et al., 2011). In addition, ESCRT was also shown to be implicated in the repair of the plasma membrane and the nuclear envelope (Jimenez et al., 2014; Lefebvre et al., 2018). The ESCRT machinery is a multipurpose toolbox within which ESCRT-III serves as the major membrane-remodeling component (Christ et al., 2017). A previous study identified that TSG101, an ESCRT-I component, could mediate the recruitment of ESCRT-III complex subunits (Radulovic et al., 2018), indicating the importance of ESCRT-I in membrane scission. VPS28 is a subunit of the ESCRT-I component, which is better known for its implication in *Candida albicans* virulence and HIV-1 budding (Cornet et al., 2006; Nydegger et al., 2006). Less evidence can be found regarding its involvement in tumor progression. The results of this study proved that cell migration, proliferation, and invasion abilities were suppressed, while cell apoptosis was enhanced, in response to VPS28 knockdown in breast cell lines. Although our study only focused on the effect of VPS28 on the biological functions of breast cancer cells, our observations contribute to a better understanding of VPS28’s involvement in malignant tumorigenesis. Those observations further encouraged us to explore its upstream targets in breast cancer cell lines.

A search for the upstream targets of VPS28, performed using the mirDIP, starBase, and TargetScan tools, showed that VPS28 binds to miR-491-5p. Data from the TCGA database demonstrated that miR-491-5p expression was suppressed in breast cancer tissues. Furthermore, the starBase tool showed that miR-491-5p levels were inversely correlated with the VPS28 expression. The dual luciferase reporter gene assay confirmed the negative regulation of VPS28 by miR-491-5p. Micro-RNA can act as a oncogene or tumor suppressor in various kinds of tumors. Evidence from a previous study supported the dysregulated expression of miR-491-5p in a wide range of malignant tumors. For example, the downregulated expression of miR-491-5p was found in nasopharyngeal carcinoma tissues and cell lines. This result suggests miR-491-5p may have the potential to be a therapeutic target in nasopharyngeal carcinoma (Zhang et al., 2016). The miR-491-5p expression of pancreatic cancer cells was reported to be lower than that of control cell lines (Guo et al., 2012). In addition, miR-491-5p levels were found to be suppressed in cervical cancer cells by JMJD2A (Li et al., 2019). Recent database publications demonstrated the possible mechanisms of miR-491-5p in suppressing the migration and invasion of breast cancer by targeting ZNF-703 to regulate the AKT/mTOR pathway or *via* TPX2 targeting (Tan et al., 2019; Guo et al., 2021). Consistent with results obtained from previous studies, our findings showed that miR-491-5p can act as a tumor suppressor in breast cancer and that the regulation of miR-491-5p expression could affect breast cancer progression. Additionally, this study also found that the overexpression of VPS28 can counteract the suppressive effect of miR-491-5p overexpression on breast cancer progression.

Although we have revealed the relationship between VPS28 and miR-491-5p to a certain extent through bioinformatics and basic experiments, the limitation of the article is also not to be ignored. In the early stage of the bioinformatic analysis, our results suggest that VPS28 can be highly expressed in luminal subtypes. This conclusion is also supported by the CCLE database, but we are not aware of this phenomenon. This led to the survival analysis to select all breast cancer patients instead of the luminal subtype. This creates bias. In our follow-up work, we will deeply explore the expression and survival of VPS28 and miR-491-5p in luminal subtypes, and collect clinical samples for verification.

Taken together, the *in silico* analyses outlined in this study highlight the role and function of VPS28 in breast cancer. Furthermore, we also identified miR-491-5p as a posttranscriptional regulator of VPS28 in breast cancer cells. Although there is prior evidence supporting the involvement of miR-491-5p in breast cancer, this study proposed another potential therapeutic target for breast cancer treatment. More studies are required to fully understand the effect of VPS28 and miR-491-5p on breast cancer progression.

## Data Availability

The original contributions presented in the study are included in the article/Supplementary Material; further inquiries can be directed to the corresponding authors.
